# Longitudinal assessment of plasma biomarkers for early detection of cognitive changes in subjective cognitive decline

**DOI:** 10.3389/fnagi.2024.1389595

**Published:** 2024-05-17

**Authors:** Cheng-Hao Hsieh, Chien-An Ko, Chih-Sung Liang, Po-Kuan Yeh, Chia-Kuang Tsai, Chia-Lin Tsai, Guan-Yu Lin, Yu-Kai Lin, Ming-Chen Tsai, Fu-Chi Yang

**Affiliations:** ^1^Department of Neurology, Tri-Service General Hospital, National Defense Medical Center, Taipei, Taiwan; ^2^Department of Psychiatry, Beitou Branch, Tri-Service General Hospital, National Defense Medical Center, Taipei, Taiwan; ^3^Department of Neurology, Songshan Branch, Tri-Service General Hospital, National Defense Medical Center, Taipei, Taiwan

**Keywords:** Alzheimer’s disease, dementia, biomarkers, neurodegenerative diseases, plasma, cognitive decline

## Abstract

**Background:**

Individuals experiencing subjective cognitive decline (SCD) are at an increased risk of developing mild cognitive impairment and dementia. Early identification of SCD and neurodegenerative diseases using biomarkers may help clinical decision-making and improve prognosis. However, few cross-sectional and longitudinal studies have explored plasma biomarkers in individuals with SCD using immunomagnetic reduction.

**Objective:**

To identify plasma biomarkers for SCD.

**Methods:**

Fifty-two participants [38 with SCD, 14 healthy controls (HCs)] underwent baseline assessments, including measurements of plasma Aβ_42_, Aβ_40_, t-tau, p-tau, and α-synuclein using immunomagnetic reduction (IMR) assays, cognitive tests and the Mini-Mental State Examination (MMSE). Following initial cross-sectional analysis, 39 individuals (29 with SCD, 10 HCs) entered a longitudinal phase for reassessment of these biomarkers and the MMSE. Biomarker outcomes across different individual categories were primarily assessed using the area under the receiver operating characteristic (ROC) curve. The SCD subgroup with an MMSE decline over one point was compared to those without such a decline.

**Results:**

Higher baseline plasma Aβ_1-42_ levels significantly discriminated participants with SCD from HCs, with an acceptable area under the ROC curve (AUC) of 67.5% [95% confidence interval (CI), 52.7–80.0%]. However, follow-up and changes in MMSE and IMR data did not significantly differ between the SCD and HC groups (*p* > 0.05). Furthermore, lower baseline plasma Aβ_1-42_ levels were able to discriminate SCD subgroups with and without cognitive decline with a satisfied performance (AUC, 75.0%; 95% CI, 55.6–89.1%). At last, the changes in t-tau and Aβ_42_ × t-tau could differentiate between the two SCD subgroups (*p* < 0.05).

**Conclusion:**

Baseline plasma Aβ_42_ may help identify people with SCD and predict SCD progression. The role of plasma Aβ_42_ levels as well as their upward trends from baseline in cases of SCD that progress to mild cognitive impairment and Alzheimer’s disease require further investigation.

## 1 Introduction

Many people seek medical advice regarding subjectively impaired cognitive function. For example, while a person may have difficulty recalling names or frequently misplace items, their performance in cognitive neuropsychological tests and daily functioning does not indicate objective cognitive impairment. Because the demand for medical guidance and support for such concerns is increasing, the term subjective cognitive decline (SCD) was introduced in 2014 (Jessen et al., 2014). The prevalence of SCD in the general adult population is 10.4–14.3% (Taylor et al., 2022). The SCD criteria comprise self-perceived persistent impairment in cognitive ability with normal performance on standardized tests to identify mild cognitive impairment (MCI), adjusted for age, sex, and education. The absence of objective cognitive impairment distinguishes SCD from MCI (Jessen et al., 2020).

SCD has attracted attention because it is associated with elevated risk of developing MCI and progression to dementia (Slot et al., 2019). A longitudinal study of 4-year follow-up data found that 27% of individuals with SCD developed MCI, and 14% of individuals with SCD developed dementia (Mitchell et al., 2014). The revised National Institute on Aging and Alzheimer’s Association research guidelines for Alzheimer’s disease (AD) classify SCD as a distinct transitional phase between asymptomatic (stage 1) and symptomatic (stage 3) MCI; thus, SCD is considered clinical stage 2 on the AD continuum (Jack et al., 2018).

SCD represents an unspecific syndrome characterized by multiple potential etiologies, including AD, minor psychiatric disorders, other comorbidities, sleep disturbances, stress, and medication use. Consequently, SCD cannot be uniformly considered synonymous with the prodromal phase of AD (Cheng et al., 2017). Individuals presenting with cognitive complaints alongside concurrent AD-associated pathological changes constitute a distinct group. This group is at an elevated risk for future cognitive decline and warrants investigative differentiation and targeted intervention. Therefore, our research is centered on the pathophysiology of AD dementia and its associated biomarkers to enhance the identification of this target group. Under normal physiological conditions, amyloid-β (Aβ) peptides are released from transmembrane amyloid precursor protein, and phosphorylated tau (p-tau) proteins maintain axonal structures. However, abnormal Aβ monomer accumulation leads to plaque formation in AD. Hyperphosphorylated tau protein causes axon degeneration, p-tau release, and subsequent neurofibrillary tangle formation (Lewczuk et al., 2020). AD is associated with reduced Aβ_1-42_ levels in cerebrospinal fluid (CSF) and elevated total tau (t-tau) levels in CSF determined using enzyme-linked immunosorbent and other conventional immunoassays (Formichi et al., 2006; Brier et al., 2016). A longitudinal study that measured CSF Aβ, t-tau, and p-tau found that low Aβ_42_ levels in CSF predict clinical progression in people with SCD (van Harten et al., 2013).

Sampling CSF is uncomfortable, invasive, and carries a minor possibility of adverse effects, especially for older adults. In contrast, blood sampling presents a less invasive option and is more feasible for widespread clinical application. However, while blood sampling allows for the detection of many serum biomarkers, the concentration of AD biomarkers in blood is typically at the picogram level, posing significant challenges for accurate measurement. To accurately detect SCD biomarkers at their low concentrations in blood, the implementation of ultrasensitive technologies like single-molecule array technology, immunomagnetic reduction (IMR), and the Meso Scale diagnostic assay is critical. These methodologies enhance the detection capabilities for AD biomarkers, making early diagnosis across the AD continuum more achievable. The principle of the IMR assay lies in eliciting a reduced response through the interaction between antibody-coated magnetic nanoparticles and target biomolecules, enabling the ultrasensitive detection of pathological plasma proteins at lower limits (Yang et al., 2008; Lue et al., 2019; Yang et al., 2020). Indeed, IMR detected higher plasma Aβ_1-42_ and t-tau levels in patients with AD or MCI than in healthy individuals (Chiu et al., 2012; Lue et al., 2019; Lee et al., 2022). These findings suggest that detection of plasma biomarkers could help determine an individual’s status on the AD continuum sooner, assess the severity of neurodegenerative diseases, or monitor disease progression (Tsai et al., 2019; Liang et al., 2020; Tsai et al., 2020; Wilczyńska and Waszkiewicz, 2020; Liang et al., 2021; Lee et al., 2022).

Recent studies have progressively elucidated the intricate relationship between SCD and AD through the lens of blood biomarkers, offering new insights into early detection and monitoring. The selection of blood biomarkers and the application of ultrasensitive assays for the exploration of SCD exhibit significant variability across different studies (Yu et al., 2023). Within the framework of “amyloid, tau, neurodegeneration” for AD pathology, potential blood-based biomarkers encompass amyloid and tau proteins, alongside indicators of neurodegeneration like neurofilament light chain (NfL) and glial fibrillary acidic protein (GFAP) (Hardy-Sosa et al., 2022; Georgakas et al., 2023).

Regarding Aβ biomarkers, the plasma Aβ_42_/Aβ_40_ ratio was highlighted in research for its efficacy in prescreening for Alzheimer’s pathology among those with SCD (Verberk et al., 2018; Palmqvist et al., 2019; Keshavan et al., 2021; Gerards et al., 2022; Zhang et al., 2024). Additionally, plasma Aβ levels might be able to successfully discriminate amyloid-positive from amyloid-negative subjects within the SCD cohort (Pan et al., 2022; Hong et al., 2023). Furthermore, a non-linear trajectory of plasma Aβ_42_ across the Alzheimer’s continuum is mapped out by one study, suggesting its predictive efficacy varies through different cognitive stages (Pan et al., 2022). Concerning p-tau biomarkers, plasma p-tau181 has been shown by numerous studies to accurately distinguish between SCD patients with and without Alzheimer’s disease pathology (Karikari et al., 2020; Palmqvist et al., 2021; Thomas et al., 2021; Gerards et al., 2022; Giacomucci et al., 2023). Moreover, plasma p-tau217 accurately distinguishes AD from other neurodegenerative conditions and substantially improves Alzheimer’s disease dementia prediction (Palmqvist et al., 2020, 2021; Janelidze et al., 2021). With respect to neurodegeneration biomarkers, plasma NfL has showed promise as a biomarker for early AD neurodegeneration (Bangen et al., 2021; Gerards et al., 2022; Giacomucci et al., 2022). Additionally, plasma GFAP levels, distinguishing dementia risk from depression in non-demented individuals, also demonstrate potential as a marker for early dementia detection (Perna et al., 2023). The integration of multiple categories of biomarkers has been strategically designed to investigate SCD. For instance, a combination of plasma Aβ_42_/Aβ_40_ and GFAP has been demonstrated to effectively identify amyloid PET status in individuals with SCD (Verberk et al., 2020). Similarly, combining plasma biomarkers Aβ_42_/Aβ_40_, P-tau217, and NfL also accurately forecast cognitive decline and AD progression in cognitively unimpaired older people (Cullen et al., 2021). Overall, the literature presents inconsistent results regarding blood biomarker profiles in individuals with SCD, and there is a paucity of data concerning the longitudinal relationship between plasma biomarkers identified through IMR and SCD individuals.

Our research is primarily focused on the following biomarkers: Aβ_42_, Aβ_40_, t-tau, p-tau181, and α-synuclein. They have been chosen for their significant association with AD and are fundamental to the pathological characterization of AD and play a crucial role in the neurodegenerative processes leading to cognitive decline. Utilizing IMR technology, this study endeavored to quantify these biomarkers in individuals experiencing SCD compared with healthy controls (HCs). Additionally, through longitudinal analysis, our objective was to clarify the predictive capacity of these biomarkers in delineating the progression from SCD to MCI and ultimately, AD. The objective of this study was twofold: first, to validate the efficacy of selected plasma biomarkers in differentiating individuals with SCD from HCs in a cross-sectional and longitudinal perspective; and second, to explore the predictive value of these biomarkers in future cognitive decline within the SCD cohort.

## 2 Materials and methods

### 2.1 Participant recruitment

This cross-sectional study recruited 52 participants from the neurology outpatient clinic at the Tri-Service General Hospital of the National Defense Medical Center, Taiwan. The criteria for diagnosing and including individuals with SCD were derived from the following established findings (Jessen et al., 2014): self-reported experience of ongoing decline in memory compared with a previous state within the past 5 years validated by information, standard performance on the Mini-Mental State Examination (MMSE) and the Montreal Cognitive Assessment (adjusted for age, sex, and education), and a score of zero in the clinical dementia rating scale. Volunteers without depression, anxiety disorders, or any conditions in the Diagnostic and Statistical Manual of Mental Disorders, Fifth Edition were recruited. Participants’ scores on standard neuropsychological tests were measured, and they were matched for age, sex, and handedness.

Participants had to have not taken medications, particularly selective serotonin reuptake inhibitors or serotonin-norepinephrine reuptake inhibitors and any other neurological medications, for a minimum of 3 months to eliminate potential effects of medication and hormone levels. All participants received a thorough medical examination and then were individually assessed by one clinical neuropsychologist at Tri-Service General Hospital. This assessment included a standardized clinical review of medical history, psychiatric and neurological examinations, physical assessments, laboratory tests for creatinine, fasting blood sugar, free thyroxine 4, high-sensitivity thyroid-stimulating hormone, vitamin B12, folic acid, serological tests for syphilis, blood parameters (numbers of platelets, white, and red blood cells, hemoglobin, mean corpuscular volume, mean corpuscular hemoglobin), and neuroimaging.

Objective cognitive performance was evaluated using standard neuropsychological tests of memory, executive functioning, and processing speed. Memory function was assessed using the 15 Word Learning Test (Brand and Jolles, 1985) and the delayed recall segment of the Rey-Osterrieth Complex Figure Test (Shin et al., 2006). Working memory was evaluated using forward and backward digit spans (Wambach et al., 2011). Executive function was assessed using the Visual Elevator (Robertson et al., 1996), Brixton Spatial Anticipation (Burgess and Shallice, 1996), and verbal fluency tests (Wilkins et al., 1987). Processing speed was assessed using a digital symbol substitution test (Jaeger, 2018) and a trail-making test (Llinàs-Reglà et al., 2017).

Additional clinical data were assessed, including the demographic factors of sex and age, cognitive assessments such as the MMSE and the clinical dementia rating scale, and supplementary measures encompassing the Pittsburgh Sleep Quality Index (Buysse et al., 1989), Beck’s Depression Inventory score (Beck et al., 1961), hospital anxiety and depression subscale score (Zigmond and Snaith, 1983), and SCD duration.

Exclusion criteria were applied uniformly to HCs and participants with SCD as follows: age < 40 or > 75 years, concurrent uncontrolled medical conditions, such as sepsis or poorly managed diabetes (hemoglobin A1c > 8.5); malignancy within the past 2 years; heart failure, myocardial infarction within the past 6 months, chronic obstructive pulmonary disease, liver cirrhosis, or renal failure; history of cerebrovascular or other neurological diseases such as neurodegeneration, epilepsy, head injury, or any Diagnostic and Statistical Manual of Mental Disorders, Fifth Edition disorder; prolonged use of psychotropic medications (serotonin-norepinephrine reuptake inhibitors, selective serotonin reuptake inhibitors, and benzodiazepines) for >6 months or substance abuse; and any structural lesion identified by brain imaging.

### 2.2 Study design

Our experimental design was divided into two segments: an initial cross-sectional component followed by a longitudinal phase. Initially, a total of 52 participants were enrolled, comprising 38 individuals with SCD and 14 HCs. Upon successful enrollment, all participants immediately underwent an initial blood draw, cognitive tests and the first MMSE to establish baseline data. The blood samples were analyzed by IMR assay for biomarkers including Plasma Aβ_42_, Aβ_40_, t-tau, p-tau181, and α-synuclein. These baseline data underwent cross-sectional analysis.

**Figure 1:**
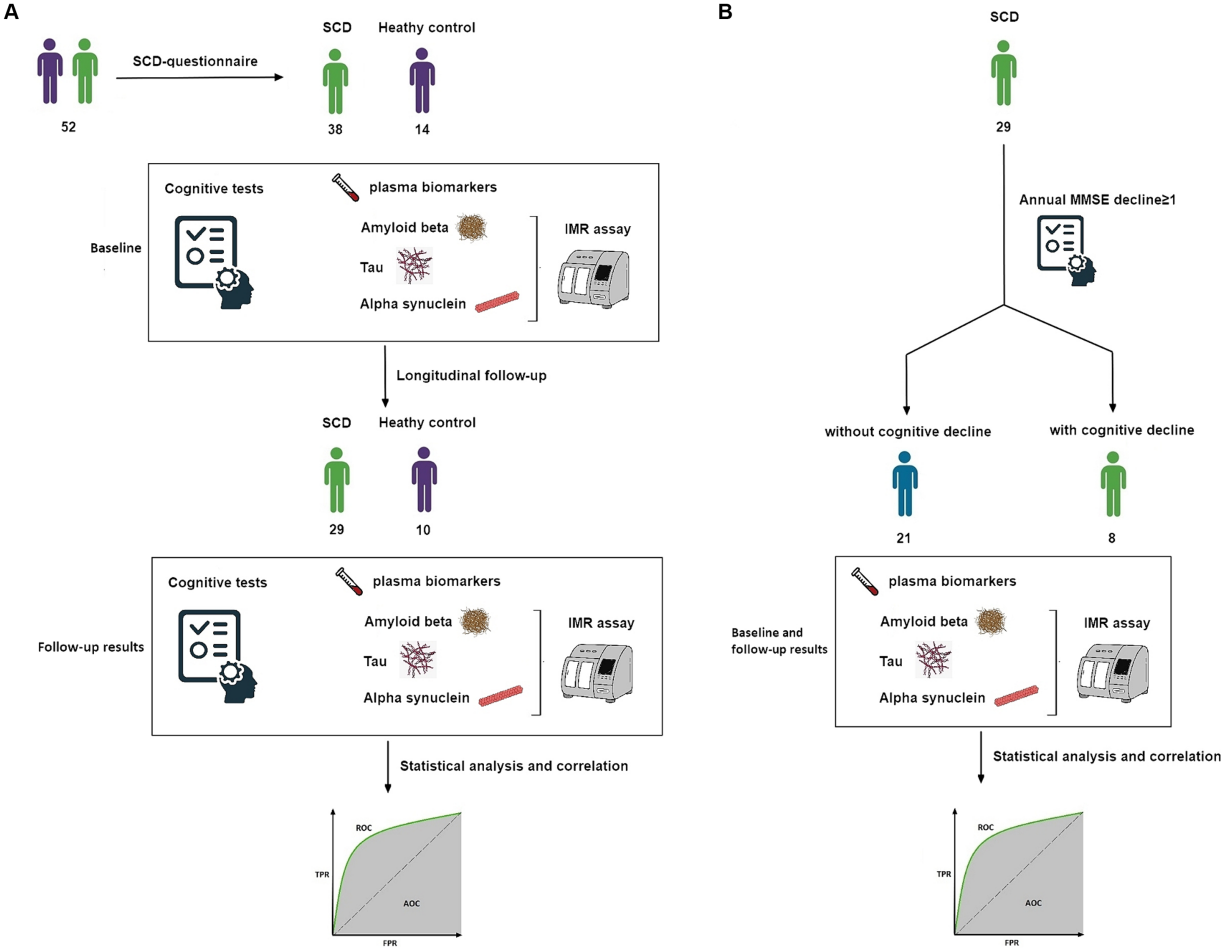
**(A)** Study design. Flow of participants through our longitudinal analysis. Data were analyzed twice. **(B)** Subgroup study design. Flow of participants with SCD and longitudinal follow-up. AUC, area under the ROC curve; IMR, immunomagnetic reduction; MMSE, Mini-Mental State Examination; ROC, receiver operating characteristic; SCD, subjective cognitive decline.

Subsequently, 39 participants continued into the longitudinal phase of the study, with a designated follow-up timeframe within 2 years, aiming to identify early correlations between cognitive decline and biomarkers. Among these, 29 were individuals with SCD and 10 were HCs, with average follow-up durations of 1.7 years and 1.2 years, respectively. All participants in this longitudinal cohort were subjected to a second blood draw and MMSE immediately after the follow-up period to gather longitudinal data. Similar to the baseline, the blood samples, including plasma Aβ_42_, Aβ_40_, t-tau, p-tau181, and α-synuclein, were assayed using the IMR method. These data were used for subsequent statistical analyses between the SCD and HC groups (Figure 1A).

Finally, among the 29 SCD participants in the longitudinal phase, individuals exhibiting an annual MMSE decline greater than one point were categorized as a group at risk of future cognitive deterioration. Conversely, those not meeting this criterion were considered unlikely to experience cognitive decline. These two subgroups underwent further analysis (Figure 1B).

### 2.3 Ethics approval and consent to participate

Written informed consent was obtained from each participant before starting the study. The Institutional Review Board of Tri-Service General Hospital approved the study (TSGHIRB 1–107–05-111).

### 2.4 Preparation of plasma samples

Non-fasting blood samples collected into 9-mL K3-EDTA tubes (455,036, Greiner Bio-one GmbH, Kremsmünster, Austria) were immediately inverted gently three times, then centrifuged at 2,300 × g for 10 min at 4°C in a swinging bucket rotor (5202R, Eppendorf AG., Hamburg, Germany). Aliquots (0.4 mL) of plasma were stored within 8 h of blood collection at −80°C in fresh 2.0 mL CryzoTraq tubes (Ziath, Cambridge, United Kingdom).

### 2.5 Plasma biomarker assays

We assayed plasma levels of Aβ_40_, Aβ_42_, t-tau, and p-tau181 using MF-AB0-0060, MF-AB2-0060, MF-TAU-0060, and MF-PT1-0060 IMR kits and a XacPro-S IMR analyzer (all from MagQu Co., New Taipei City, Taiwan). The protocol for these assays mandates combining 40 μL of plasma for Aβ_40_, t-tau, and p-tau181 tests, as opposed to 60 μL of plasma for the Aβ_42_ test, with their corresponding reagents, resulting in a total reaction volume of 120 μL. The discrepancy in plasma volume requirements for Aβ_40_ and Aβ_42_ assays is attributable to their differential baseline concentrations; the lower concentration of Aβ_42_ relative to Aβ_40_ necessitates the use of a larger plasma volume for Aβ_42_ to ensure the accuracy and precision of the assay. Results are shown as the means of duplicate measurements. The reliable ranges for detecting Aβ_40_, Aβ_42_, t-tau, and p-tau181 in the IMR assay were 0.17–1,000, 0.77–30,000, 0.026–3,000, and 0.0196–1,000 pg./mL, respectively. The intra- or inter-assay coefficients of variation for Aβ_40_, Aβ_42_, t-tau, or p-tau181 assays using IMR were 7–10% and 10–15% for high- and low-concentration quality control samples, respectively. We used two different batches of reagents per biomarker with variations in reagent properties between batches maintained at <10%. We applied rigorous quality control measures to ensure consistent particle size, concentrations, and bioactivity.

### 2.6 Statistical analysis

We compared demographics, cognitive test outcomes, and IMR data in paired groups (SCD vs. HCs; cognitive decline vs. maintenance) using independent sample t-tests for continuous variables or Fisher’s exact tests for categorical variables. We compared MMSE and IMR data and further adjusted age and education levels using a multivariable linear regression analysis. The ability of baseline IMR data to discriminate participants with SCD from HCs was assessed by analyzing receiver operating characteristic (ROC) curves. The ability of baseline, follow-up, and changes in IMR data to discriminate cognitive decline (annual MMSE decline ≥1) among participants with SCD was also evaluated using the area under the ROC curve (AUC). The confidence interval (CI) of the AUC was calculated using the DeLong nonparametric method. All tests were 2-tailed, and *p* < 0.05 was considered significant. Data were analyzed using SPSS version 26 (IBM SPSS Inc., Chicago, Illinois, United States).

## 3 Results

### 3.1 Inclusion, demographics, clinical, and study characteristics

Table 1 shows the baseline clinical and demographic information, cognitive test results, and IMR data of the 14 HCs and 38 participants with SCD. The demographic characteristics of age, sex, education level, and body mass index did not significantly differ between the groups. The results of cognitive tests and baseline MMSE scores also did not significantly differ between the groups. Both groups harbored notably similar frequencies of the APOE ε4 allele, which is the major genetic risk factor for placement on the AD continuum.

**Table 1:** Demographics, cognitive, and IMR findings at baseline for healthy controls and individuals with SCD.

Variable	Control (*n* = 14)	SCD (*n* = 38)	*p*	*p* ^*^
Demographics				
Age, y	67.6 ± 8.9	68.3 ± 8.7	0.806	–
Male (%)	3 (21.4)	14 (36.8)	0.341	–
Education level, y	11.6 ± 3.8	10.3 ± 4.9	0.373	–
Body mass index, kg/m^2^	25.8 ± 4.7	24.3 ± 3.5	0.206	–
Cognitive tests				
tCDR	0.6 ± 0.6	0.8 ± 0.8	0.437	–
Disease index	11.4 ± 0.9	11.0 ± 1.1	0.191	–
fDS	10.5 ± 2.6	10.9 ± 2.3	0.548	–
bDS	7.4 ± 3.6	6.3 ± 2.8	0.290	–
VFT	13.6 ± 2.9	13.4 ± 3.0	0.793	–
TMTA	51.6 ± 34.0	51.6 ± 18.6	0.990	–
Baseline MMSE	28.4 ± 1.3	27.7 ± 2.0	0.249	0.366
Baseline IMR data (pg/mL)				
t-tau	22.8 ± 3.3	25.5 ± 6.7	0.151	0.134
Aβ_42_	16.4 ± 0.9	17.2 ± 1.1	0.035	0.026
p-tau181	3.8 ± 0.7	4.1 ± 0.8	0.314	0.328
Aβ_40_	53.0 ± 5.7	51.4 ± 4.5	0.308	0.293
α-synuclein (fg/mL)	154.4 ± 135.3	126.1 ± 105.4	0.435	0.453
Aβ_42_ × t-tau	375.6 ± 66.2	445.0 ± 148.9	0.101	0.087
Aβ_42_ × p-tau181	62.8 ± 12.9	70.5 ± 18.9	0.168	0.169
Aβ_42_/Aβ_40_	0.31 ± 0.04	0.34 ± 0.04	0.079	0.069
*APOE* ε4 allele frequency	3 (23.1)	6 (16.2)	0.679	–

Data are presented as means ± standard deviations or as frequencies (%). ^*^Adjusted for age and education level. Aβ, amyloid-β; APOE, apolipoprotein E; bDS, backward digit span; fDS, forward digit span; HVLT, Hopkins Verbal Learning Test; IMR, ultra-sensitive immunomagnetic reduction; MMSE, Mini-Mental Status Examination; p-tau181, tau phosphorylated at threonine 181; SCD, subjective cognitive decline; tCDR, total score of Clinical Dementia Rating; TMTA, Trail Making Test Part A; t-tau, total tau; VFT, verbal fluency test.

**Figure 2:**
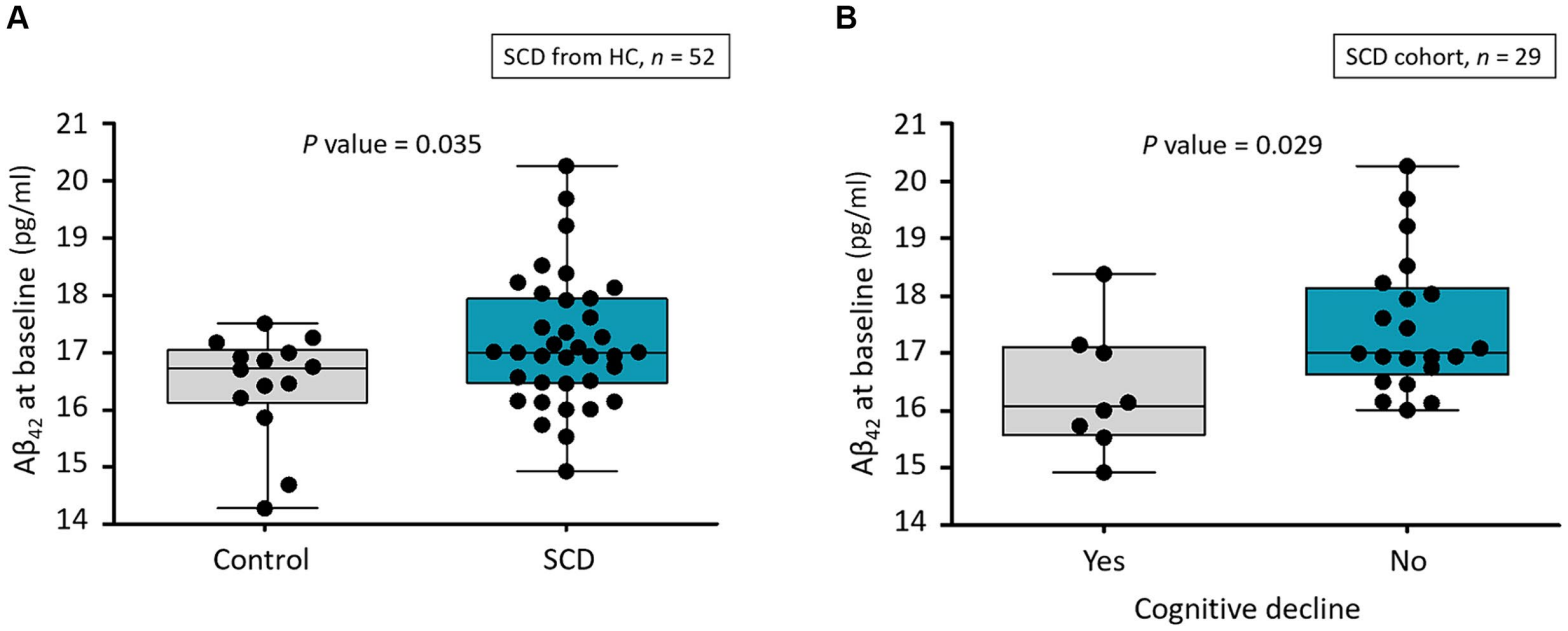
**(A)** Distribution of Aβ_42_ levels at baseline in healthy controls and participants with SCD. Aβ, amyloid-β; HC, healthy control; SCD, subjective cognitive decline. **(B)** Distribution of Aβ_42_ level at baseline for participants who had SCD with and without cognitive decline as annual MMSE decline of ≥1. Aβ, amyloid-β; MMSE, Mini-Mental Status Examination; SCD, subjective cognitive decline.

Among the plasma biomarkers, only baseline levels of Aβ_42_ were significantly higher in the SCD group than in HCs (17.2 ± 1.1 vs. 16.4 ± 0.9 pg./mL, *p* = 0.035; Table 1 and Figure 2A). This finding persisted after adjusting for age and educational level (*p* = 0.026). The AUC for Aβ_42_ to discriminate SCD from HC was 67.5% (95% CI, 52.7–80.0%; Supplementary Files S1, S4). All plasma biomarker levels were within their respective detection limits using IMR.

### 3.2 Longitudinal changes in MMSE and IMR data and correlations between groups during follow-up

We assessed annual changes and follow-up data including MMSE scores and plasma biomarker levels to identify longitudinal associations between plasma biomarkers and cognitive decline. We compared these data in 10 HCs and 29 participants with SCD who had complete follow-up MMSE and IMR data for 1.2 and 1.7 years, respectively. Follow-up and changes in MMSE and IMR data did not significantly differ between the SCD and HC groups (Table 2) and were consistent after adjusting for age and educational level.

**Table 2:** Changes in MMSE and IMR data in healthy controls and individuals with SCD during follow-up.

Variable	Control	SCD	*p*	*p* ^*^
Duration, year	1.2 ± 0.4	1.7 ± 0.7	0.065	–
Follow-up MMSE	28.0 ± 1.5	28.0 ± 2.7	0.969	0.923
Follow-up IMR data (pg/mL)				
t-tau	22.1 ± 3.0	23.7 ± 3.7	0.230	0.268
Aβ_42_	16.5 ± 0.8	16.9 ± 0.9	0.142	0.177
p-tau181	3.9 ± 0.4	3.7 ± 0.7	0.544	0.559
Aβ_40_	48.2 ± 7.6	49.1 ± 6.6	0.749	0.707
α-synuclein (fg/mL)	82.7 ± 39.2	107.6 ± 50.3	0.170	0.173
Aβ_42_ × t-tau	363.5 ± 53.1	402.9 ± 75.8	0.142	0.176
Aβ_42_ × p-tau181	63.9 ± 6.5	63.6 ± 13.7	0.947	0.924
Aβ_42_/Aβ_40_	0.35 ± 0.07	0.35 ± 0.05	0.956	0.970
Annual MMSE change	−0.3 ± 1.4	0.3 ± 1.7	0.351	0.432
Change in IMR data (pg/mL)				
t-tau	0.3 ± 3.5	−2.6 ± 8.5	0.308	0.355
Aβ_42_	0.2 ± 1.1	−0.3 ± 1.5	0.394	0.438
p-tau181	0.2 ± 0.7	−0.4 ± 1.3	0.155	0.214
Aβ_40_	−4.4 ± 11.1	−1.8 ± 7.7	0.434	0.457
α-synuclein (fg/mL)	−29.3 ± 52.8	−30.5 ± 138.0	0.978	0.955
Aβ_42_ × t-tau	8.6 ± 73.3	−58.5 ± 186.4	0.280	0.319
Aβ_42_ × p-tau181	3.2 ± 14.3	−9.4 ± 26.6	0.167	0.223
Aβ_42_/Aβ_40_	0.04 ± 0.09	0.01 ± 0.06	0.277	0.306

Data are presented as means ± standard deviations or frequency (%). ^*^Adjusted for age and education level. Aβ, amyloid-β; IMR, ultra-sensitive immunomagnetic reduction; MMSE, Mini-Mental Status Examination; p-tau181, tau phosphorylated at threonine 181; SCD, subjective cognitive decline; t-tau, total tau.

### 3.3 Associations between baseline plasma biomarkers and risk of cognitive decline in individuals with SCD

We assigned the follow-up data of the 29 participants with SCD to two subgroups to determine longitudinal correlations between SCD and subsequent corresponding cognitive decline. Among the 29 individuals, 8 (27.6%) were considered to have subsequent cognitive decline. Only baseline Aβ_42_ significantly differed among demographics, cognitive test findings, MMSE score, and all IMR data (Table 3). The baseline Aβ_42_ level was significantly lower in the individuals with than without subsequent cognitive decline (16.4 ± 1.1 vs. 17.5 ± 1.2 pg./mL, *p* = 0.029; Figure 2B). This result remained significant after adjusting for age and educational level (*p* = 0.048). The AUC for Aβ_42_ to discriminate subsequent cognitive decline among participants with SCD was 75.0% (95% CI, 55.6–89.1%; Supplementary File S2 and Table 4).

**Table 3:** Baseline demographics, cognitive, and IMR findings of participants with SCD with/without cognitive decline.

Variable	Declined (*n* = 8)	Maintained (*n* = 21)	*p*	*p* ^*^
Demographics				
Age, y	65.5 ± 8.0	69.6 ± 8.3	0.245	–
Male	3 (37.5)	6 (28.6)	0.675	–
Education level,	11.1 ± 4.3	10.5 ± 5.0	0.765	–
Body mass index, kg/m^2^	24.0 ± 4.1	24.0 ± 3.1	0.998	–
Cognitive tests				
tCDR	0.6 ± 0.4	0.9 ± 0.8	0.427	–
HVLT	21.3 ± 2.7	22.8 ± 3.8	0.299	–
fDS	11.6 ± 2.6	11.1 ± 2.1	0.605	–
bDS	7.8 ± 3.3	6.2 ± 2.4	0.187	–
VFT	13.6 ± 2.6	13.3 ± 3.3	0.797	–
TMTA	49.3 ± 17.5	49.7 ± 15.2	0.944	–
Baseline MMSE	27.8 ± 2.9	27.4 ± 1.8	0.682	0.739
Baseline IMR data (pg/mL)				
t-tau	21.9 ± 4.8	26.8 ± 7.4	0.097	0.131
Aβ_42_	16.4 ± 1.1	17.5 ± 1.2	0.029	0.048
p-tau181	3.9 ± 0.7	4.2 ± 0.9	0.511	0.617
Aβ_40_	49.9 ± 4.9	51.6 ± 4.4	0.356	0.313
α-synuclein (fg/mL)	98.0 ± 57.3	144.8 ± 130.9	0.342	0.416
Aβ_42_ × t-tau	363.2 ± 103.5	475.9 ± 167.9	0.089	0.120
Aβ_42_ × p-tau181	64.6 ± 14.8	73.6 ± 21.8	0.293	0.378
Aβ_42_/Aβ_40_	0.33 ± 0.05	0.34 ± 0.04	0.636	0.804
ApoE ε4 allele frequency	1 (12.5)	4 (20.0)	1.000	–

Data are presented as means ± standard deviations or frequency (%). ^*^Adjusted for age and education level. Aβ, amyloid-β; APOE, apolipoprotein E; bDS, backward digit span; fDS, forward digit span; HVLT, Hopkins Verbal Learning Test; IMR, ultra-sensitive immunomagnetic reduction; MMSE, Mini-Mental Status Examination; p-tau, tau phosphorylated at threonine 181; SCD, subjective cognitive decline; tCDR, total score of Clinical Dementia Rating; TMTA, Trail Making Test Part A; t-tau, total tau; VFT, verbal fluency test.

**Table 4:** Ability of baseline, follow-up, and changes in IMR data to discriminate annual MMSE decline ≥1 among the individuals with SCD.

	Baseline		Follow-up		Change	
IMR data	AUC (95% CI)	*P*	AUC (95% CI)	*p*	AUC (95% CI)	*p*
t-tau	69.6 (49.9–85.2)	0.098	50.8 (30.0–71.2)	0.953	75.4 (54.2–90.2)	0.028
Aβ_42_	75.0 (55.6–89.1)	0.042	67.5 (46.0–84.7)	0.164	65.1 (43.6–82.9)	0.222
p-tau181	54.2 (34.8–72.7)	0.723	58.7 (37.5–77.8)	0.527	51.6 (30.9–71.8)	0.905
Aβ_40_	57.7 (38.1–75.7)	0.523	67.5 (46.0–84.7)	0.141	69.0 (47.6–85.8)	0.075
α-synuclein	64.9 (45.0–81.6)	0.218	54.8 (33.8–74.6)	0.732	61.9 (40.5–80.4)	0.348
Aβ_42_ × t-tau	71.4 (51.7–86.5)	0.071	54.8 (33.8–74.6)	0.707	75.4 (54.2–90.2)	0.028
Aβ_42_ × p-tau181	57.7 (38.1–75.7)	0.518	62.7 (41.3–81.0)	0.327	54.0 (0.33–0.74)	0.758
Aβ_42_ / Aβ_40_	56.0 (36.4–74.2)	0.666	67.5 (46.0–84.7)	0.174	56.3 (35.2–75.9)	0.618

Aβ, amyloid-β; AUC, area under the ROC curve; CI, confidence interval; IMR, ultra-sensitive immunomagnetic reduction; MMSE, Mini-Mental Status Examination; p-tau181, tau phosphorylated at threonine 181; ROC, receiver operating characteristic; SCD, subjective cognitive decline; t-tau, total tau.

### 3.4 Changes and associations in plasma biomarkers at follow-up and risk of cognitive decline in individuals with SCD

Our assessment revealed no significant differences in follow-up and changes in IMR data between the subgroups of patients with SCD (Supplementary File S5). However, the AUC indicated that changes in t-tau and Aβ42 × t-tau could differentiate between the two subgroups (Supplementary File S3 and Table 4). Changes in t-tau and Aβ_42_ × t-tau were higher in the subgroup with subsequent cognitive decline.

## 4 Discussion

This IMR-based study found that increased levels of baseline plasma Aβ_42_ significantly discriminated individuals with SCD from HCs. The measured concentration in this study (17.2 ± 1.1 pg./mL) was higher than the reported level in HCs and is close to the reported level in amnestic mild cognitive impairment (aMCI) measured by several groups independently (Tzen et al., 2014; Lue et al., 2017; Chen et al., 2019; Chiu et al., 2020; Jiao et al., 2020; Tsai et al., 2020; Hu et al., 2021), as shown in Table 5. This implies that amyloidosis is occurring in the brain at the stage of SCD, prior to MCI. Interventions or treatments should be given to individuals with SCD to prevent or delay the onset of MCI or AD. Although people with SCD do not show cognitive impairment as significant as that observed in aMCI, similar plasma Aβ_42_ levels are detected in individuals with SCD and aMCI. This indicates that abnormal levels of plasma Aβ_42_ are detectable prior to the onset of clinically diagnosed cognitive impairment. Plasma biomarkers are promising for use as preclinical assessments for future AD.

**Table 5:** Plasma Aβ_42_ concentrations measured using IMR in HC, SCD, and aMCI reported by other groups and in the current study.

No.	HC	SCD	aMCI	Ref.
1	16.6 ± 0.7	–	17.0 ± 0.7	Tsai et al. (2020)
2	15.9 ± 0.3	–	17.2 ± 0.3	Tzen et al. (2014)
3	15.33 ± 0.66	–	–	Lue et al. (2017)
4	–	–	17.3 - 20.1	Chen et al. (2019)
5	15.8 ± 0.7	–	17.6 ± 1.8	Chiu et al. (2020)
6	16.92 ± 1.67	–	–	Jiao et al. (2020)
7	15.71 ± 2.13	–	–	Hu et al. (2021)
8	16.4 ± 0.9	17.2 ± 1.1	–	This work

Aβ, amyloid-β; aMCI, amnestic mild cognitive impairment; HC, healthy control; IMR, ultra-sensitive immunomagnetic reduction; SCD, subjective cognitive decline.

Based on previous studies (Chiu et al., 2013, 2020), plasma Aβ_42_ × t-tau is suggested to be a powerful index for assessing aMCI and AD. Furthermore, plasma Aβ_42_ × t-tau predicts the progression to MCI in HCs. According to Lee et al. (2023), 20% of cognitively normal individuals with baseline plasma Aβ_42_ × t-tau levels higher than 382 (pg/mL)^2^ progress to MCI in 1 to 2 years. Notably, the measured plasma Aβ_42_ × t-tau levels in individuals with SCD in this study were 445.0 ± 148.9 (pg/mL)^2^. Most individuals with SCD are at risk for cognitive decline in the near future. The 1.7-year longitudinal observations of participants with SCD showed that 8 of 29 of these individuals (27.6%) experienced further cognitive decline. Consistent results obtained between these studies demonstrate the feasibility of predicting future cognitive decline in cognitively normal subjects using plasma Aβ_42_ × t-tau.

In Table 3, lower baseline plasma Aβ_42_ levels significantly discriminated participants in the SCD subgroups with real cognitive decline (16.4 ± 1.1 pg./mL) from those with maintained cognitive function (17.5 ± 1.2 pg./mL, *p* < 0.05). From the point of view of AD progression, the levels of plasma Aβ_42_ assayed with IMR continuously elevate from HC to SCD and aMCI to AD. Presumably, individuals with higher levels of plasma Aβ_42_ are at a relatively severe stage of AD and should have rapid progression in cognitive decline. However, the results in Table 3 show that individuals with SCD with relatively low levels of Aβ_42_ already display real cognitive decline. The results are opposite to the expectation. Chi et al. reported similar results in post-stroke cognitive impairment (PSCI) (Chi et al., 2019; Huang et al., 2022). Patients diagnosed with PSCI 1 year after stroke had lower Aβ_42_ levels (15.1 pg./mL) at 3 months than those without PSCI (17.2 pg./mL, p < 0.05). The downstream pathogenesis underlying changes in plasma Aβ_42_ in individuals with SCD or PSCI are unclear. Degradation of the glymphatic system or meningeal lymphatic vessels in the brain may cause poor clearance of Aβ_42_ from the brain to peripheral blood, thereby contributing to the relatively low plasma Aβ_42_ levels (Chi et al., 2019; Huang et al., 2022). Meanwhile, more Aβ_42_ accumulates in the brain, resulting in the rapid decline in cognition in individuals with SCD or PSCI. More investigations are needed to clarify the detailed downstream mechanism underlying changes in plasma Aβ_42_ levels in individuals with SCD or PSCI displaying real decline in cognition.

Changes in t-tau and Aβ_42_ × t-tau might discriminate subsequent cognitive decline among individuals with SCD. This means that plasma t-tau is a key biomarker reflecting the severity of disease. A negative correlation (*r* = −0.93, *p* < 0.05) between the changes in plasma t-tau levels and changes in MMSE scores in aMCI has been reported (Tsai et al., 2020). Furthermore, several studies show significant changes in plasma t-tau levels in participants at high risk of AD after intervention (Lin and Li, 2022; Su et al., 2022; Te Liu et al., 2023). All the results suggest plasma t-tau is a promising biomarker for monitoring the progression/conversion of cognitive impairment.

Changes in plasma Aβ levels throughout the progression of cognitive decline along the AD continuum are uneven and unpredictable (Pan et al., 2022). The present findings show that higher baseline plasma Aβ_42_ levels significantly discriminated patients with SCD from HCs. These were consistent with previous findings indicating that plasma Aβ_42_ could serve as a preliminary screening tool for detecting AD-related pathological changes in healthy individuals experiencing SCD (De Rojas et al., 2018; Verberk et al., 2018; Gerards et al., 2022; Pan et al., 2022). Our findings could assist clinicians in identifying individuals with SCD among healthy populations. Blood tests are less invasive and more readily accessible than CSF tests and serve as vital references and a foundation for clinicians to conduct thorough assessments and formulate medical plans.

AD may stem from increased production or compromised clearance of Aβ_42_, leading to the deposition of amyloid plaques (Potter et al., 2013). The involved clearance systems, namely the blood–brain barrier, glymphatic system, and meningeal lymphatic vessels, are likely to become dysfunctional, thus contributing to impaired Aβ removal from the brain to the bloodstream (Tarasoff-Conway et al., 2015). This is expressed as declining plasma and CSF Aβ_42_ concentrations (Lewczuk et al., 2020). A conceivable explanation for the increased plasma Aβ_42_ level in SCD might be that clearance systems in preclinical AD are not yet compromised, and Aβ_42_ transport from the brain to the bloodstream is augmented.

Plasma Aβ_42_ values increase from normal cognition to SCD on the AD continuum, followed by a decline in MCI and AD (Pan et al., 2022). The non-linear nature of plasma Aβ throughout the AD continuum might explain our findings. Specifically, baseline plasma Aβ_42_ levels were obviously lower in individuals with SCD who experienced subsequent cognitive decline compared with those who had preserved cognitive function. Nonetheless, participants with SCD were distinguished from HCs by significantly elevated baseline plasma Aβ_42_ levels.

Tau protein predominantly localizes in the central nervous system, where it facilitates crucial microtubule assembly and stabilization. Tau is hyperphosphorylated in people on the AD continuum, and it aggregates to form neurofibrillary tangles that have neurotoxic effects on neurons. The transmission of these pathological forms of tau among neurons might contribute to the progressive spread of AD within the brain (Guo and Lee, 2011; Medina and Avila, 2014). Our findings indicate that altered t-tau levels or product of Aβ_42_ × t-tau levels could differentiate persons with SCD who develop subsequent cognitive decline from those who do not. These results suggest that fluctuations in tau levels could serve as prospective biomarkers along the AD continuum, given that t-tau is a biomarker of neurodegeneration (Holper et al., 2022). Nonetheless, the significance of the data lacks reproducibility across statistical methodologies, specifically between independent sample t-tests and ROC analyses. Consequently, the strength of evidence derived from these findings is comparatively diminished compared with those pertaining to baseline plasma Aβ_1-42_ levels. Therefore, alterations in t-tau levels hold great promise as prognostic, rather than diagnostic biomarkers.

In our quest to refine diagnostic approaches for the earliest stages of the Alzheimer’s continuum, our study has embraced plasma biomarkers because of their less invasive collection method and greater accessibility to clinicians. This choice aims to facilitate broader clinical application in anticipation of the upcoming era of disease-modifying therapies for AD. However, a critical concern emerges during the transition of biomarker analysis from CSF to plasma. While CSF biomarkers demonstrate pronounced differences between individuals, plasma biomarkers reveal only subtle absolute variations across different patient categories. These minimal variations are susceptible to distortion by preanalytical and analytical confounders, thereby diminishing their discriminative power, as evidenced by the AUC values. Moreover, CSF biomarkers offer more of an AD pathology landscape through the A/T/N system. Incorporating CSF biomarkers enables us to better distinguish SCD patients with an underlying Alzheimer’s pathology from those without. This capability suggests a promising avenue for future research to further explore plasma biomarkers by correlating them with CSF biomarkers, potentially enhancing our understanding and identification of AD at its nascent stages.

Despite the remarkable progress in blood biomarker research facilitated by ultra-sensitive assays in recent years, their integration into clinical practice demands rigorous validation and standardization. This challenge is further compounded by factors such as individual preferences, healthcare economics, and cultural perceptions of aging (Georgakas et al., 2023). The advent of disease-modifying treatments offers compelling motivation for advancing blood biomarker research in the near future. Future research is essential not only to develop biomarkers that offer greater accuracy and reliability but also to deepen our understanding of the insights these biomarkers yield. Additionally, it is crucial to investigate the potential for testing these biomarkers directly in whole blood. Grasping this comprehensive knowledge is imperative for influencing patient healthcare decisions, clinical outcomes, and overall well-being.

### 4.1 Limitations

This study of correlations between biomarkers in individuals with SCD and HCs has some limitations. The sample size was relatively modest, necessitating the need for larger-scale studies in the future. Furthermore, future studies should broaden the scope beyond the examination of amyloid and tau proteins to encompass additional markers, such as neurofilament light chain and glial fibrillary acidic protein.

In conclusion, we found that elevated baseline plasma Aβ_1-42_ levels can discriminate individuals with SCD from HCs, which is a valuable insight for clinicians. Diminished baseline plasma Aβ_1-42_ levels have discriminatory potential for identifying cognitive deterioration among patients with SCD. The discriminatory roles of changes in t-tau levels or the product of Aβ_42_ × t-tau levels were similar. Identifying SCD with these biomarkers could facilitate the early detection of neurodegenerative diseases and thus lead to timely intervention. The mechanisms underlying the course of plasma Aβ level changes from elevation to decline during the AD continuum should also be investigated.

## Data availability statement

The raw data supporting the conclusions of this article will be made available by the authors, without undue reservation.

## Ethics statement

The studies involving humans were approved by The Institutional Review Board of Tri-Service General Hospital (TSGHIRB 1–107–05-111). The studies were conducted in accordance with the local legislation and institutional requirements. The participants provided their written informed consent to participate in this study.

## Author contributions

C-HH: Conceptualization, Data curation, Formal analysis, Investigation, Project administration, Validation, Visualization, Writing – original draft, Writing – review & editing. C-AK: Data curation, Validation, Writing – review & editing. C-SL: Data curation, Investigation, Visualization, Writing – review & editing. P-KY: Data curation, Validation, Writing – review & editing. C-KT: Data curation, Visualization, Writing – review & editing. C-LT: Validation, Writing – review & editing. G-YL: Data curation, Validation, Writing – review & editing. Y-KL: Data curation, Investigation, Writing – review & editing. M-CT: Data curation, Writing – review & editing. F-CY: Conceptualization, Data curation, Formal analysis, Funding acquisition, Investigation, Methodology, Project administration, Resources, Software, Supervision, Validation, Visualization, Writing – original draft, Writing – review & editing.

## References

[ref1] Bangen K. J. Thomas K. R. Weigand A. J. Edmonds E. C. Clark A. L. Solders S.. (2021). Elevated plasma neurofilament light predicts a faster rate of cognitive decline over 5 years in participants with objectively-defined subtle cognitive decline and MCI. Alzheimers Dement. 17, 1756–1762. doi: 10.1002/ALZ.12324, PMID: 33860596 PMC8517034

[ref2] Beck A. T. Ward C. H. Mendelson M. Mock J. Erbaugh J. (1961). An inventory for measuring depression. Arch. Gen. Psychiatry 4, 561–571. doi: 10.1001/ARCHPSYC.1961.01710120031004 13688369

[ref3] Brand N. Jolles J. (1985). Learning and retrieval rate of words presented auditorily and visually. J. Gen. Psychol. 112, 201–210. doi: 10.1080/00221309.1985.9711004, PMID: 4056765

[ref4] Brier M. R. Gordon B. Friedrichsen K. McCarthy J. Stern A. Christensen J.. (2016). Tau and ab imaging, CSF measures, and cognition in Alzheimer’s disease. Sci. Transl. Med. 8:338ra66. doi: 10.1126/scitranslmed.aaf2362, PMID: 27169802 PMC5267531

[ref5] Burgess P. W. Shallice T. (1996). Bizarre responses, rule detection and frontal lobe lesions. Cortex 32, 241–259. doi: 10.1016/S0010-9452(96)80049-9, PMID: 8800613

[ref6] Buysse D. J. Reynolds C. F. Monk T. H. Berman S. R. Kupfer D. J. (1989). The Pittsburgh sleep quality index: a new instrument for psychiatric practice and research. Psychiatry Res. 28, 193–213. doi: 10.1016/0165-1781(89)90047-4, PMID: 2748771

[ref7] Chen T. B. Lee Y. J. Lin S. Y. Chen J. P. Hu C. J. Wang P. N.. (2019). Plasma Aβ42 and Total tau predict cognitive decline in amnestic mild cognitive impairment. Sci. Rep. 9:13984. doi: 10.1038/S41598-019-50315-9, PMID: 31562355 PMC6764975

[ref8] Cheng Y. W. Chen T. F. Chiu M. J. (2017). From mild cognitive impairment to subjective cognitive decline: conceptual and methodological evolution. Neuropsychiatr. Dis. Treat. 13, 491–498. doi: 10.2147/NDT.S123428, PMID: 28243102 PMC5317337

[ref9] Chi N. F. Chao S. P. Huang L. K. Chan L. Chen Y. R. Chiou H. Y.. (2019). Plasma amyloid Beta and tau levels are predictors of post-stroke cognitive impairment: a longitudinal study. Front. Neurol. 10:715. doi: 10.3389/FNEUR.2019.00715, PMID: 31312178 PMC6614443

[ref10] Chiu M. J. Chen T. F. Hu C. J. Yan S. H. Sun Y. Liu B. H.. (2020). Nanoparticle-based immunomagnetic assay of plasma biomarkers for differentiating dementia and prodromal states of Alzheimer’s disease - a cross-validation study. Nanomedicine 28:102182. doi: 10.1016/J.NANO.2020.102182 32222476

[ref11] Chiu M. J. Yang S. Y. Chen T. F. Chieh J. J. Huang T. Z. Yip P. K.. (2012). New assay for old markers-plasma Beta amyloid of mild cognitive impairment and Alzheimer’s disease. Curr. Alzheimer Res. 9, 1142–1148. doi: 10.2174/156720512804142967, PMID: 22950866

[ref12] Chiu M. J. Yang S. Y. Horng H. E. Yang C. C. Chen T. F. Chieh J. J.. (2013). Combined plasma biomarkers for diagnosing mild cognition impairment and Alzheimer’s disease. ACS Chem. Neurosci. 4, 1530–1536. doi: 10.1021/CN400129P, PMID: 24090201 PMC3867966

[ref13] Cullen N. C. Leuzy A. Janelidze S. Palmqvist S. Svenningsson A. L. Stomrud E.. (2021). Plasma biomarkers of Alzheimer’s disease improve prediction of cognitive decline in cognitively unimpaired elderly populations. Nat. Commun. 12, 1–9. doi: 10.1038/s41467-021-23746-0, PMID: 34117234 PMC8196018

[ref14] De Rojas I. Romero J. Rodríguez-Gomez O. Pesini P. Sanabria A. Pérez-Cordon A.. (2018). Correlations between plasma and PET beta-amyloid levels in individuals with subjective cognitive decline: the Fundació ACE healthy brain initiative (FACEHBI). Alzheimers Res. Ther. 10:119. doi: 10.1186/S13195-018-0444-1, PMID: 30497535 PMC6267075

[ref16] Formichi P. Battisti C. Radi E. Federico A. (2006). Cerebrospinal fluid tau, Aß, and phosphorylated tau protein for the diagnosis of Alzheimer’s disease. J. Cell. Physiol. 208, 39–46. doi: 10.1002/JCP.20602 16447254

[ref17] Georgakas J. E. Howe M. D. Thompson L. I. Riera N. M. Riddle M. C. (2023). Biomarkers of Alzheimer’s disease: past, present and future clinical use. Biomark Neuropsychiatry 8:100063. doi: 10.1016/J.BIONPS.2023.100063

[ref18] Gerards M. Schild A. K. Meiberth D. Rostamzadeh A. Vehreschild J. J. Wingen-Heimann S.. (2022). Alzheimer’s disease plasma biomarkers distinguish clinical diagnostic groups in memory clinic patients. Dement. Geriatr. Cogn. Disord. 51, 182–192. doi: 10.1159/000524390, PMID: 35504263

[ref19] Giacomucci G. Mazzeo S. Bagnoli S. Ingannato A. Leccese D. Berti V.. (2022). Plasma neurofilament light chain as a biomarker of Alzheimer’s disease in subjective cognitive decline and mild cognitive impairment. J. Neurol. 269, 4270–4280. doi: 10.1007/s00415-022-11055-5, PMID: 35288777 PMC9293849

[ref20] Giacomucci G. Mazzeo S. Bagnoli S. Ingannato A. Padiglioni S. Sorbi S.. (2023). Plasma p-tau181 as a promising non-invasive biomarker of Alzheimer’s disease pathology in subjective cognitive decline. Neurology 100:17. doi: 10.1212/WNL.0000000000203114 37716237

[ref21] Guo J. L. Lee V. M. Y. (2011). Seeding of normal tau by pathological tau conformers drives pathogenesis of Alzheimer-like tangles. J. Biol. Chem. 286, 15317–15331. doi: 10.1074/JBC.M110.209296, PMID: 21372138 PMC3083182

[ref22] Hardy-Sosa A. León-Arcia K. Llibre-Guerra J. J. Berlanga-Acosta J. Baez S. D. L. C. Guillen-Nieto G.. (2022). Diagnostic accuracy of blood-based biomarker panels: a systematic review. Front. Aging Neurosci. 14:683689. doi: 10.3389/fnagi.2022.683689, PMID: 35360215 PMC8963375

[ref24] Holper S. Watson R. Yassi N. (2022). Tau as a biomarker of neurodegeneration. Int. J. Mol. Sci. 23:7307. doi: 10.3390/IJMS23137307, PMID: 35806324 PMC9266883

[ref25] Hong Y. J. Ho S. H. Jeong J. H. Park K. H. Kim S. Y. Wang M. J.. (2023). Impacts of baseline biomarkers on cognitive trajectories in subjective cognitive decline: the CoSCo prospective cohort study. Alzheimers Res. Ther. 15, 1–10. doi: 10.1186/S13195-023-01273-Y/FIGURES/3 37550761 PMC10405399

[ref26] Hu C. J. Chiu M. J. Pai M. C. Yan S. H. Wang P. N. Chiu P. Y.. (2021). Assessment of high risk for Alzheimer’s disease using plasma biomarkers in subjects with Normal cognition in Taiwan: a preliminary study. J. Alzheimers Dis. Rep. 5, 761–770. doi: 10.3233/ADR-210310, PMID: 34870102 PMC8609520

[ref27] Huang L. K. Chao S. P. Hu C. J. Chien L. N. Chiou H. Y. Lo Y. C.. (2022). Plasma phosphorylated-tau181 is a predictor of post-stroke cognitive impairment: a longitudinal study. Front. Aging Neurosci. 14:889101. doi: 10.3389/FNAGI.2022.889101, PMID: 35572134 PMC9099290

[ref28] Jack C. R. Bennett D. A. Blennow K. Carrillo M. C. Dunn B. Haeberlein S. B.. (2018). NIA-AA research framework: toward a biological definition of Alzheimer’s disease. Alzheimers Dement. 14, 535–562. doi: 10.1016/J.JALZ.2018.02.018, PMID: 29653606 PMC5958625

[ref001] Jaeger J. (2018). Digit symbol substitution test: the case for sensitivity over specificity in neuropsychological testing. J. Clin. Psychopharmacol. 38:513. doi: 10.1097/JCP.0000000000000941 30124583 PMC6291255

[ref29] Janelidze S. Berron D. Smith R. Strandberg O. Proctor N. K. Dage J. L.. (2021). Associations of plasma Phospho-Tau217 levels with tau positron emission tomography in early Alzheimer disease. JAMA Neurol. 78, 149–156. doi: 10.1001/JAMANEUROL.2020.4201, PMID: 33165506 PMC7653537

[ref30] Jessen F. Amariglio R. E. Buckley R. F. van der Flier W. M. Han Y. Molinuevo J. L.. (2020). The characterisation of subjective cognitive decline. Lancet Neurol. 19, 271–278. doi: 10.1016/S1474-4422(19)30368-0, PMID: 31958406 PMC7062546

[ref31] Jessen F. Amariglio R. E. Van Boxtel M. Breteler M. Ceccaldi M. Chételat G.. (2014). A conceptual framework for research on subjective cognitive decline in preclinical Alzheimer’s disease. Alzheimers Dement. 10, 844–852. doi: 10.1016/J.JALZ.2014.01.001, PMID: 24798886 PMC4317324

[ref32] Jiao F. Yi F. Wang Y. Zhang S. Guo Y. Du W.. (2020). The validation of multifactor model of plasma Aβ 42 and Total-tau in combination with MoCA for diagnosing probable Alzheimer disease. Front. Aging Neurosci. 12:212. doi: 10.3389/FNAGI.2020.00212, PMID: 32792940 PMC7385244

[ref33] Karikari T. K. Pascoal T. A. Ashton N. J. Janelidze S. Benedet A. L. Rodriguez J. L.. (2020). Blood phosphorylated tau 181 as a biomarker for Alzheimer’s disease: a diagnostic performance and prediction modelling study using data from four prospective cohorts. Lancet Neurol. 19, 422–433. doi: 10.1016/S1474-4422(20)30071-5 32333900

[ref34] Keshavan A. Pannee J. Karikari T. K. Rodriguez J. L. Ashton N. J. Nicholas J. M.. (2021). Population-based blood screening for preclinical Alzheimer’s disease in a British birth cohort at age 70. Brain 144, 434–449. doi: 10.1093/BRAIN/AWAA403, PMID: 33479777 PMC7940173

[ref35] Lee Y. J. Lin S. Y. Peng S. W. Lin Y. C. Chen T. B. Wang P. N.. (2023). Predictive utility of plasma amyloid and tau for cognitive decline in cognitively normal adults. J. Prev Alzheimers Dis. 10, 178–185. doi: 10.14283/JPAD.2023.15, PMID: 36946444

[ref36] Lee P.-J. Tsai C.-L. Liang C.-S. Peng G. S. Lee J.-T. Tsai C.-K.. (2022). Biomarkers with plasma amyloid β and tau protein assayed by Immunomagnetic reduction in patients with amnestic mild cognitive impairment and Alzheimer’s disease. Acta Neurol. Taiwanica 31, 53–60. 35040113

[ref37] Lewczuk P. Łukaszewicz-Zając M. Mroczko P. Kornhuber J. (2020). Clinical significance of fluid biomarkers in Alzheimer’s disease. Pharmacol. Rep. 72, 528–542. doi: 10.1007/S43440-020-00107-0, PMID: 32385624 PMC7329803

[ref38] Liang C. S. Su K. P. Tsai C. L. Lee J. T. Chu C. S. Yeh T. C.. (2020). The role of interleukin-33 in patients with mild cognitive impairment and Alzheimer’s disease. Alzheimers Res. Ther. 12:86. doi: 10.1186/s13195-020-00652-z, PMID: 32678011 PMC7367330

[ref39] Liang C.-S. Tsai C.-L. Lin G.-Y. Lee J.-T. Lin Y.-K. Chu C.-S.. (2021). Better identification of cognitive decline with Interleukin-2 than with amyloid and tau protein biomarkers in amnestic mild cognitive impairment. Front. Aging Neurosci. 13:670115. doi: 10.3389/fnagi.2021.670115, PMID: 34122046 PMC8193360

[ref40] Lin L. J. Li K. Y. (2022). Comparing the effects of olfactory-based sensory stimulation and board game training on cognition, emotion, and blood biomarkers among individuals with dementia: a pilot randomized controlled trial. Front. Psychol. 13:1003325. doi: 10.3389/FPSYG.2022.1003325, PMID: 36204759 PMC9531625

[ref58] Liu W. Te Huang H. T. Hung H. Y. Lin S. Y. Hsu W. H. Lee F. Y.. (2023). Continuous positive airway pressure reduces plasma neurochemical levels in patients with OSA: a pilot study. Life 13,:613. doi: 10.3390/LIFE13030613, PMID: 36983769 PMC10059911

[ref41] Llinàs-Reglà J. Vilalta-Franch J. López-Pousa S. Calvó-Perxas L. Torrents Rodas D. Garre-Olmo J. (2017). The trail making test: association with other neuropsychological measures and normative values for adults aged 55 years and older from a Spanish-speaking population-based sample. Assessment 24, 183–196. doi: 10.1177/1073191115602552 26318386

[ref42] Lue L. F. Kuo Y. M. Sabbagh M. (2019). Advance in plasma AD Core biomarker development: current findings from immunomagnetic reduction-based SQUID technology. Neurol. Ther. 8, 95–111. doi: 10.1007/S40120-019-00167-2 PMC6908530 31833027

[ref43] Lue L. F. Sabbagh M. N. Chiu M. J. Jing N. Snyder N. L. Schmitz C.. (2017). Plasma levels of Aβ42 and tau identified probable Alzheimer’s dementia: findings in two cohorts. Front. Aging Neurosci. 9:226. doi: 10.3389/FNAGI.2017.00226, PMID: 28790911 PMC5522888

[ref44] Medina M. Avila J. (2014). The role of extracellular tau in the spreading of neurofibrillary pathology. Front. Cell. Neurosci. 8:113. doi: 10.3389/FNCEL.2014.00113, PMID: 24795568 PMC4005959

[ref45] Mitchell A. J. Beaumont H. Ferguson D. Yadegarfar M. Stubbs B. (2014). Risk of dementia and mild cognitive impairment in older people with subjective memory complaints: meta-analysis. Acta Psychiatr. Scand. 130, 439–451. doi: 10.1111/ACPS.12336, PMID: 25219393

[ref46] Palmqvist S. Janelidze S. Quiroz Y. T. Zetterberg H. Lopera F. Stomrud E.. (2020). Discriminative accuracy of plasma Phospho-tau217 for Alzheimer disease vs other neurodegenerative disorders. JAMA 324, 772–781. doi: 10.1001/JAMA.2020.12134 32722745 PMC7388060

[ref47] Palmqvist S. Janelidze S. Stomrud E. Zetterberg H. Karl J. Zink K.. (2019). Performance of fully automated plasma assays as screening tests for Alzheimer Disease–related β-amyloid status. JAMA Neurol. 76, 1060–1069. doi: 10.1001/JAMANEUROL.2019.1632, PMID: 31233127 PMC6593637

[ref48] Palmqvist S. Tideman P. Cullen N. Zetterberg H. Blennow K. Dage J. L.. (2021). Prediction of future Alzheimer’s disease dementia using plasma phospho-tau combined with other accessible measures. Nat. Med. 27, 1034–1042. doi: 10.1038/s41591-021-01348-z 34031605

[ref49] Pan F. F. Huang Q. Wang Y. Wang Y. F. Guan Y. H. Xie F.. (2022). Non-linear character of plasma amyloid beta over the course of cognitive decline in Alzheimer’s continuum. Front. Aging Neurosci. 14:832700. doi: 10.3389/FNAGI.2022.832700, PMID: 35401142 PMC8984285

[ref50] Perna L. Stocker H. Burow L. Beyer L. Trares K. Kurz C.. (2023). Subjective cognitive complaints and blood biomarkers of neurodegenerative diseases: a longitudinal cohort study. Alzheimers Res. Ther. 15, 1–12. doi: 10.1186/S13195-023-01341-3/TABLES/4 37951931 PMC10638700

[ref51] Potter R. Patterson B. W. Elbert D. L. Ovod V. Kasten T. Sigurdson W.. (2013). Increased in vivo amyloid-b42 production, exchange, and loss in presenilin mutation carriers. Sci. Transl. Med. 5:189ra77. doi: 10.1126/scitranslmed.3005615, PMID: 23761040 PMC3838868

[ref52] Robertson I. H. Ward T. Ridgeway V. Nimmo-Smith I. (1996). The structure of normal human attention: the test of everyday attention. J. Int. Neuropsychol. Soc. 2, 525–534. doi: 10.1017/S1355617700001697 9375156

[ref53] Shin M. S. Park S. Y. Park S. R. Seol S. H. Kwon J. S. (2006). Clinical and empirical applications of the Rey–Osterrieth complex Figure test. Nat. Protoc. 1, 892–899. doi: 10.1038/nprot.2006.115 17406322

[ref54] Slot R. E. R. Sikkes S. A. M. Berkhof J. Brodaty H. Buckley R. Cavedo E.. (2019). Subjective cognitive decline and rates of incident Alzheimer’s disease and non-Alzheimer’s disease dementia. Alzheimers Dement. 15, 465–476. doi: 10.1016/J.JALZ.2018.10.003, PMID: 30555032 PMC6465066

[ref55] Su M. T. Lu C. W. Wu W. J. Jheng Y. S. Yang S. Y. Chuang W. C.. (2022). Applications of Immunomagnetic reduction technology as a biosensor in therapeutic evaluation of Chinese herbal medicine in Tauopathy alleviation of an AD Drosophila model. Biosensors 12:883. doi: 10.3390/BIOS12100883, PMID: 36291020 PMC9599240

[ref56] Tarasoff-Conway J. M. Carare R. O. Osorio R. S. Glodzik L. Butler T. Fieremans E.. (2015). Clearance systems in the brain-implications for Alzheimer disease. Nat. Rev. Neurol. 11, 457–470. doi: 10.1038/NRNEUROL.2015.119, PMID: 26195256 PMC4694579

[ref57] Taylor C. A. Bouldin E. D. McGuire L. C. (2022). Subjective cognitive decline among adults aged ≥45 years — United States, 2015–2016. MMWR Morb. Mortal Wkly. Rep. 67, 753–757. doi: 10.15585/MMWR.MM6727A1, PMID: 30001562 PMC6047468

[ref59] Thomas K. R. Bangen K. J. Edmonds E. C. Weigand A. J. Walker K. S. Bondi M. W.. (2021). Objective subtle cognitive decline and plasma phosphorylated tau181: early markers of Alzheimer’s disease-related declines. Alzheimer’s Dement. 13:e12238. doi: 10.1002/DAD2.12238 PMC8515224 34692978

[ref60] Tsai C. L. Liang C. S. Lee J. T. Su M. W. Lin C. C. Chu H.. (2019). Associations between plasma biomarkers and cognition in patients with Alzheimer’s disease and amnestic mild cognitive impairment: a cross-sectional and longitudinal study. J. Clin. Med. 8,:1893. doi: 10.3390/JCM8111893, PMID: 31698867 PMC6912664

[ref61] Tsai C. L. Liang C. S. Yang C. P. Lee J. T. Ho T. H. Su M. W.. (2020). Indicators of rapid cognitive decline in amnestic mild cognitive impairment: the role of plasma biomarkers using magnetically labeled immunoassays. J. Psychiatr. Res. 129, 66–72. doi: 10.1016/j.jpsychires.2020.06.006 32592947

[ref62] Tzen K. Y. Yang S. Y. Chen T. F. Cheng T. W. Horng H. E. Wen H. P.. (2014). Plasma Aβ but not tau is related to brain PiB retention in early Alzheimer’s disease. ACS Chem. Neurosci. 5, 830–836. doi: 10.1021/CN500101J, PMID: 25054847

[ref63] van Harten A. C. Visser P. J. Pijnenburg Y. A. L. Teunissen C. E. Blankenstein M. A. Scheltens P.. (2013). Cerebrospinal fluid Aβ42 is the best predictor of clinical progression in patients with subjective complaints. Alzheimers Dement. 9, 481–487. doi: 10.1016/J.JALZ.2012.08.004, PMID: 23232269

[ref64] Verberk I. M. W. Slot R. E. Verfaillie S. C. J. Heijst H. Prins N. D. van Berckel B. N. M.. (2018). Plasma amyloid as Prescreener for the earliest Alzheimer pathological changes. Ann. Neurol. 84, 648–658. doi: 10.1002/ANA.25334, PMID: 30196548 PMC6282982

[ref65] Verberk I. M. W. Thijssen E. Koelewijn J. Mauroo K. Vanbrabant J. De Wilde A.. (2020). Combination of plasma amyloid beta(1-42/1-40)and glial fibrillary acidic protein strongly associates with cerebral amyloid pathology. Alzheimers Res. Ther. 12, 1–14. doi: 10.1186/S13195-020-00682-7/FIGURES/5 PMC7523295 32988409

[ref002] Wambach D. Lamar M. Swenson R. Penney D. L. Kaplan E. Libon D. J. (2011). Digit Span. Encycl. Clin. Neuropsychol. 844–849. doi: 10.1007/978-0-387-79948-3_1288

[ref66] Wilczyńska K. Waszkiewicz N. (2020). Diagnostic utility of selected serum dementia biomarkers: amyloid β-40, amyloid β −42, tau protein, and YKL-40: a review. J. Clin. Med. 9, 1–26. doi: 10.3390/jcm9113452, PMID: 33121040 PMC7692800

[ref67] Wilkins A. J. Shallice T. McCarthy R. (1987). Frontal lesions and sustained attention. Neuropsychologia 25, 359–365. doi: 10.1016/0028-3932(87)90024-8 3601041

[ref68] Yang S. Y. Jian Z. F. Horng H. E. Hong C. Y. Yang H. C. Wu C. C.. (2008). Dual immobilization and magnetic manipulation of magnetic nanoparticles. J. Magn. Magn. Mater. 320, 2688–2691. doi: 10.1016/J.JMMM.2008.05.048

[ref69] Yang S. Y. Liu H. C. Chen W. P. (2020). Immunomagnetic reduction detects plasma Aβ1-42 levels as a potential dominant Indicator predicting cognitive decline. Neurol. Ther. 9, 435–442. doi: 10.1007/S40120-020-00215-2, PMID: 33090326 PMC7606390

[ref70] Yu X. Shao K. Wan K. Li T. Li Y. Zhu X.. (2023). Progress in blood biomarkers of subjective cognitive decline in preclinical Alzheimer’s disease. Chin. Med. J. 136, 505–521. doi: 10.1097/CM9.0000000000002566, PMID: 36914945 PMC10106168

[ref71] Zhang M. Wang X. Zhao W. Li Y. Ying C. Jiang J.. (2024). Subjective cognitive decline domain improves accuracy of plasma Aβ42/Aβ40 for preclinical Alzheimer’s disease diagnosis: the SILCODE study. Chin. Med. J. 137:1127. doi: 10.1097/CM9.0000000000002851, PMID: 37946327 PMC11062658

[ref72] Zigmond A. S. Snaith R. P. (1983). The hospital anxiety and depression scale. Acta Psychiatr. Scand. 67, 361–370. doi: 10.1111/J.1600-0447.1983.TB09716.X 6880820

