# Supplementary figures and images for: Longitudinal assessment of plasma biomarkers for early detection of cognitive changes in subjective cognitive decline
Source: Front Aging Neurosci. 2024 May 17;16:1389595. doi: 10.3389/fnagi.2024.1389595 (PMC11140011; doi:10.3389/fnagi.2024.1389595)

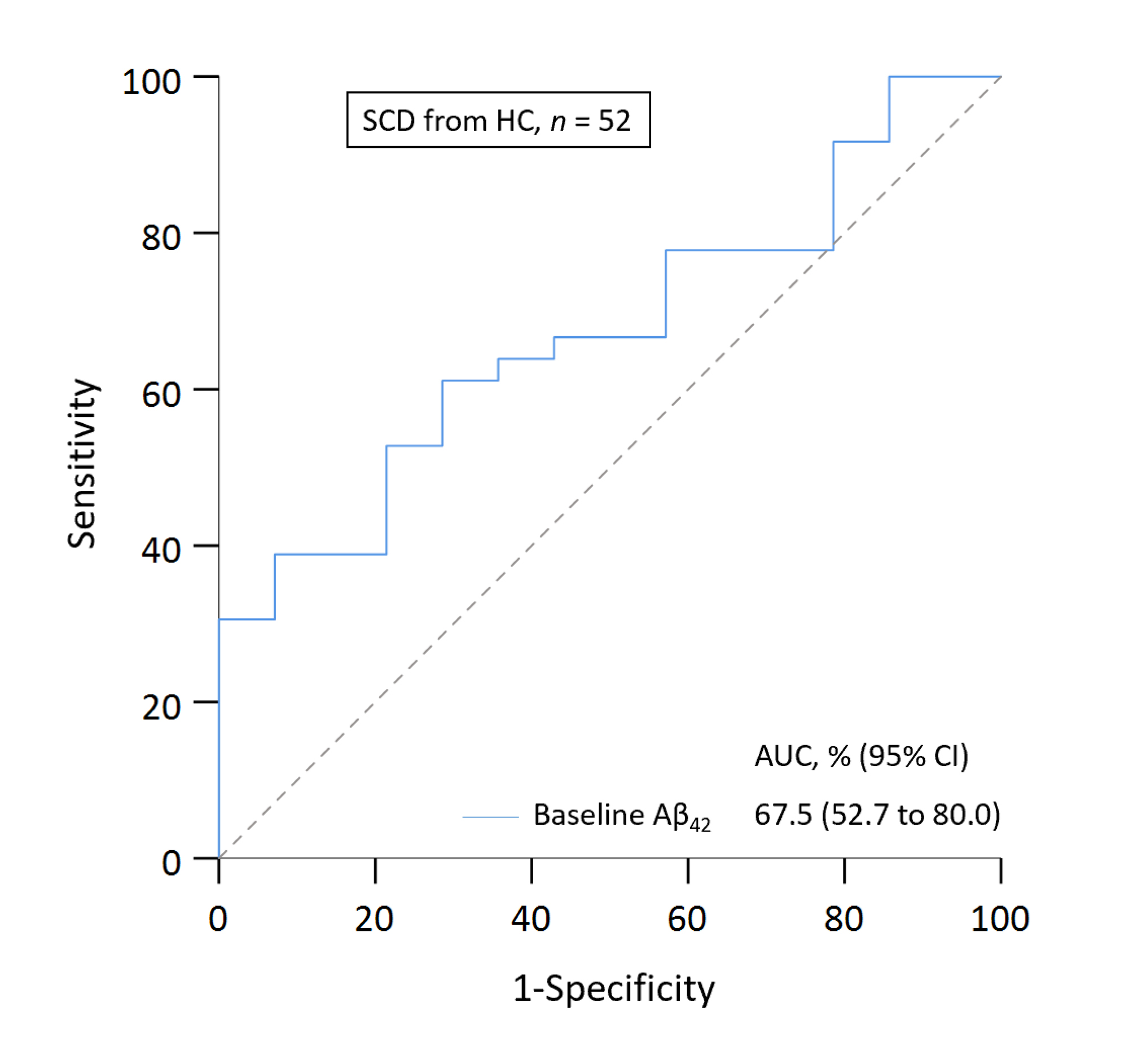

Supplement: Supplementary file 1 [file Image_1.JPEG]

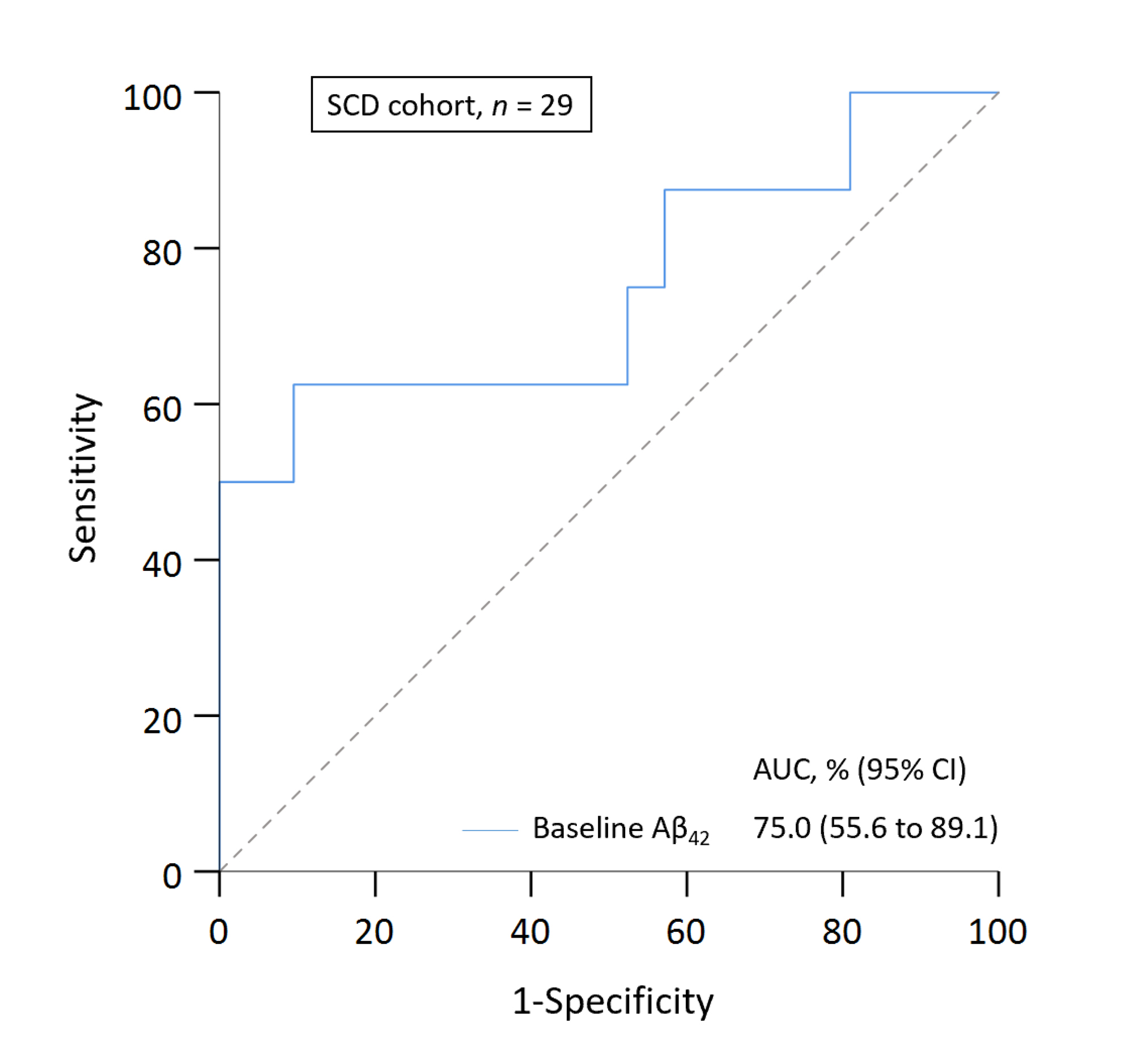

Supplement: Supplementary file 2 [file Image_2.JPEG]

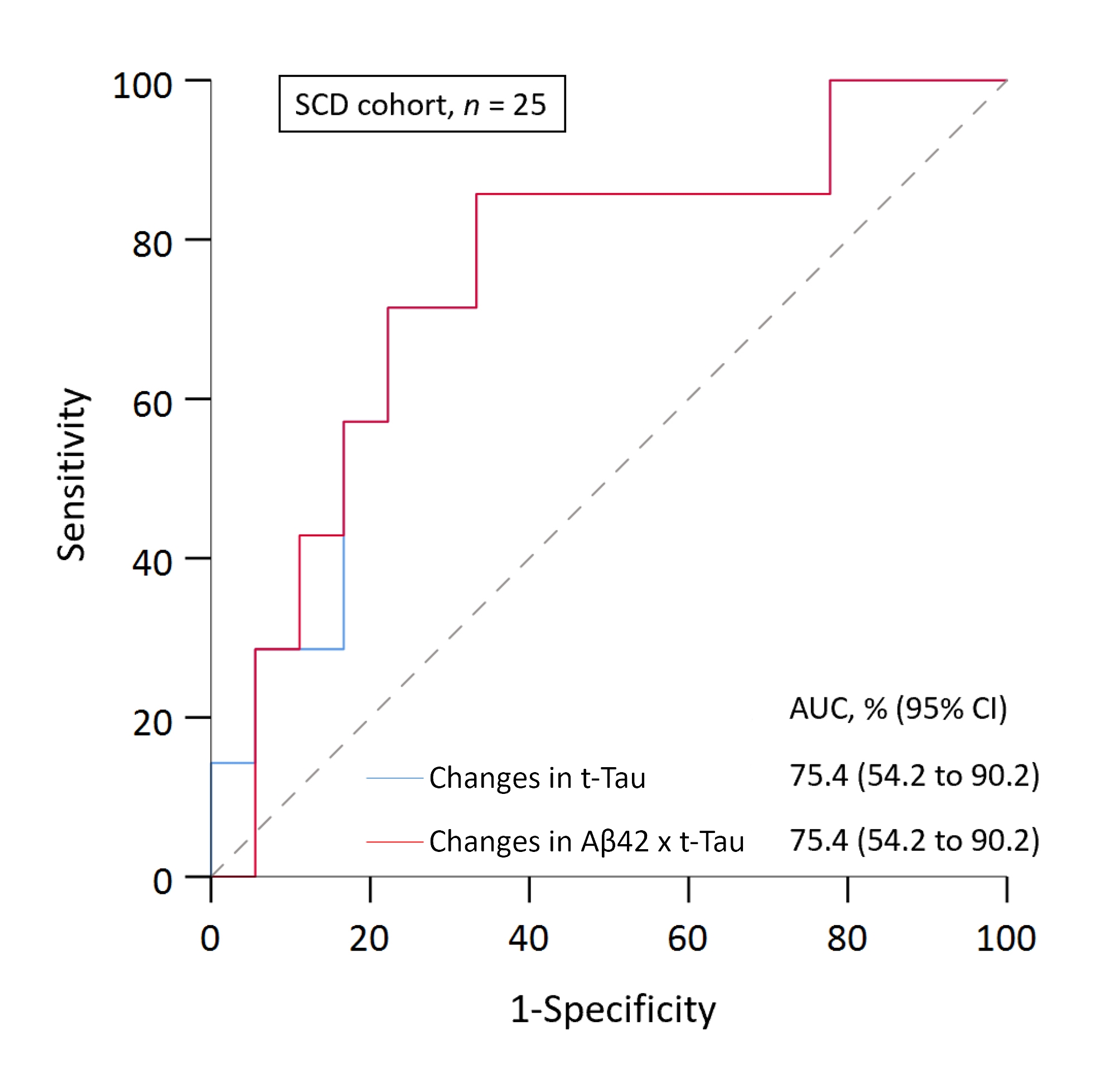

Supplement: Supplementary file 3 [file Image_3.JPEG]
