# Supplementary material for: Longitudinal assessment of plasma biomarkers for early detection of cognitive changes in subjective cognitive decline
Source: Front Aging Neurosci. 2024 May 17;16:1389595. doi: 10.3389/fnagi.2024.1389595 (PMC11140011; doi:10.3389/fnagi.2024.1389595)
Supplement: Supplementary file 4 [file Table_1.DOCX]

Supplementary Material

**Supplemental Table 1**. The ability of the baseline IMR data to discriminate patients with SCD from healthy controls

| IMR data | AUC, % (95% CI) | *P* value |
| --- | --- | --- |
| t-tau | 60.5 (45.7 to 74.0) | 0.211 |
| Aβ_42_ | 67.5 (52.7 to 80.0) | 0.028 |
| p-tau181 | 58.1 (43.3 to 71.9) | 0.384 |
| Aβ_40_ | 57.1 (42.4 to 71.0) | 0.466 |
| α-synuclein | 59.5 (44.7 to 73.2) | 0.316 |
| Aβ_42_ × t-tau | 64.7 (50.0 to 77.7) | 0.073 |
| Aβ_42_ × p-tau181 | 61.3 (46.5 to 74.7) | 0.229 |
| Aβ_42_ / Aβ_40_ | 62.3 (47.5 to 75.6) | 0.182 |

Abbreviations: IMR, ultra-sensitive immunomagnetic reduction; SCD, subjective cognitive decline; AUC, area under the curve; CI, confidence interval; t-tau, total Tau; Aβ, amyloid-β; p-tau181, tau phosphorylated at threonine181
