# Supplementary material for: Longitudinal assessment of plasma biomarkers for early detection of cognitive changes in subjective cognitive decline
Source: Front Aging Neurosci. 2024 May 17;16:1389595. doi: 10.3389/fnagi.2024.1389595 (PMC11140011; doi:10.3389/fnagi.2024.1389595)
Supplement: Supplementary file 5 [file Table_2.DOCX]

Supplementary Material

**Supplemental Table 2**. Follow-up and changes in MMSE and IMR data of patients with SCD with/without cognitive decline

| Variable | Decline  (*n* = 8) | Maintain  (*n* = 21) | *P* value | *P* value^*^ |
| --- | --- | --- | --- | --- |
| Duration, year | 1.6 ± 0.8 | 1.7 ± 0.6 | 0.698 | - |
| Follow-up MMSE | 25.4 ± 4.0 | 29.0 ± 1.1 | <0.001 | 0.001 |
| Follow-up IMR data |  |  |  |  |
| t-tau (pg/mL) | 23.9 ± 3.6 | 23.8 ± 3.9 | 0.954 | 0.996 |
| Aβ_42_ (pg/mL) | 16.5 ± 0.6 | 17.1 ± 0.9 | 0.147 | 0.116 |
| p-tau181 (pg/mL) | 3.6 ± 0.6 | 3.8 ± 0.8 | 0.591 | 0.450 |
| Aβ_40_ (pg/mL) | 51.7 ± 7.0 | 48.3 ± 6.4 | 0.262 | 0.321 |
| α-synuclein (fg/mL) | 106.9 ± 44.4 | 104.1 ± 52.4 | 0.904 | 0.967 |
| Aβ_42_ × t-tau | 395.1 ± 61.7 | 409.0 ± 82.7 | 0.692 | 0.630 |
| Aβ_42_ × p-tau181 | 60.1 ± 8.7 | 65.7 ± 15.2 | 0.369 | 0.261 |
| Aβ_42_ / Aβ_40_ | 0.3 ± 0.0 | 0.4 ± 0.1 | 0.125 | 0.125 |
| Annual MMSE change | -1.6 ± 1.2 | 1.0 ± 1.2 | <0.001 | <0.001 |
| Change of IMR data |  |  |  |  |
| t-tau (pg/mL) | 2.8 ± 7.1 | -3.9 ± 8.1 | 0.068 | 0.104 |
| Aβ_42_ (pg/mL) | 0.3 ± 1.3 | -0.5 ± 1.6 | 0.289 | 0.425 |
| p-tau181 (pg/mL) | -0.2 ± 1.0 | -0.4 ± 1.3 | 0.724 | 0.837 |
| Aβ_40_ (pg/mL) | 2.1 ± 4.5 | -3.1 ± 8.5 | 0.141 | 0.176 |
| α-synuclein (fg/mL) | 9.6 ± 77.0 | -50.6 ± 156.4 | 0.345 | 0.389 |
| Aβ_42_ × t-tau | 48.0 ± 140.5 | -86.1 ± 187.1 | 0.101 | 0.150 |
| Aβ_42_ × p-tau181 | -3.5 ± 19.4 | -10.1 ± 29.1 | 0.590 | 0.716 |
| Aβ_42_ / Aβ_40_ | -0.01 ± 0.05 | 0.02 ± 0.06 | 0.427 | 0.385 |

Abbreviations: MMSE, Mini-Mental Status Examination; IMR, ultra-sensitive immunomagnetic reduction; SCD, subjective cognitive decline; t-tau, total tau; Aβ, amyloid-β; p-tau181, tau phosphorylated at threonine 181

Data are presented as mean ± standard deviation.

^*^Adjusted for age and education level.
